# Heart-specific DNA methylation analysis in plasma for the investigation of myocardial damage

**DOI:** 10.1186/s12967-022-03234-9

**Published:** 2022-01-21

**Authors:** Jie Ren, Lin Jiang, Xiaomeng Liu, Yuhan Liao, Xueyan Zhao, Fuchou Tang, Huimin Yu, Yibing Shao, Jizheng Wang, Lu Wen, Lei Song

**Affiliations:** 1 Biomedical Pioneering Innovation Center, School of Life Sciences, Ministry of Education Key Laboratory of Cell Proliferation and Differentiation, Beijing, 100871 China; 2 State Key Laboratory of Cardiovascular Disease, Fuwai Hospital, National Center for Cardiovascular Diseases, Chinese Academy of Medical Sciences and Peking Union Medical College, Beijing, 100871 China; 3 Beijing Advanced Innovation Center for Genomics, Peking University, Beijing, 100871 China; 4 Peking-Tsinghua Center for Life Sciences, Academy for Advanced Interdisciplinary Studies, Peking University, Beijing, 100871 China; 5 National Clinical Research Center for Cardiovascular Diseases, Fuwai Hospital, National Center for Cardiovascular Diseases, Chinese Academy of Medical Sciences and Peking Union Medical College, Beijing, 100871 China; 6 Department of Cardiology, Guangdong Cardiovascular Institute, Guangdong Provincial People’s Hospital, Guangdong Academy of Medical Sciences, Guangzhou, China; 7 Department of Cardiology, Qingdao Municipal Hospital, Qingdao, Shandong China; 8 Radiation Oncology Key Laboratory of Sichuan Province, Sichuan Cancer Hospital & Institute, Sichuan Cancer Center, School of Medicine, University of Electronic Science and Technology of China, Chengdu, China

**Keywords:** Circulating cell-free DNA, DNA methylation, Myocardial infarction, Sequencing, ddPCR

## Abstract

**Background:**

Circulating cell-free DNA (cfDNA) can be released when myocardial damage occurs.

**Methods:**

Here, we used the methylated CpG tandem amplification and sequencing (MCTA-seq) method for analyzing dynamic changes in heart-derived DNA in plasma samples from myocardial infarction (MI) patients.

**Results:**

We identified six CGCGCGG loci showing heart-specific hypermethylation patterns. MCTA-seq deconvolution analysis combining these loci detected heart-released cfDNA in MI patients at hospital admission, and showed that the prominently elevated total cfDNA level after percutaneous coronary intervention (PCI) was derived from both the heart and white blood cells. Furthermore, for the top marker *CORO6*, we developed a digital droplet PCR (ddPCR) assay that clearly detected heart damage signals in cfDNA of MI patients at hospital admission.

**Conclusions:**

Our study provides insights into MI pathologies and developed a new ddPCR assay for detecting myocardial damage in clinical applications.

**Supplementary Information:**

The online version contains supplementary material available at 10.1186/s12967-022-03234-9.

## Background

Circulating cell-free DNA (cfDNA) is emerging as a powerful tool for diagnosing and monitoring diseases. It has been successfully used in clinical practice for noninvasive prenatal testing and liquid biopsy for cancer, and its utility in graft rejection is being investigated [1–6]. However, these genetic-based approaches are not applicable for situations where cfDNA originates from tissues with a normal genome. DNA methylation, a stable tissue-specific epigenetic modification, has recently been investigated for assessing the tissue of origin of cfDNA. We and others have established deconvolution methods for tissue fractions using whole-genome and target-enrichment DNA methylation methods [7–13]. In addition, single-marker assays for a variety of tissues, including the pancreas, brain, liver, colon, and heart, have been reported [8, 13–15].

Cardiovascular diseases, including myocardial infarctions (MIs), are the leading causes of death worldwide. MIs are known to be associated with cell death. A previous study has shown that the concentration of cfDNA is elevated in MI patients, with series sampling showing that the cfDNA level usually peaks later than creatine kinase-MB (CK-MB), but the source of the increased cfDNA is not clearly understood [16]. In a recent landmark study, Zemmour et al. [14] has shown that DNA from dying cardiomyocytes can be released into the blood as cfDNA. The marker FAM101A has been reported to be a cardiomyocyte-specific unmethylated marker which increases in the plasma of MI patients. However, no heart-specific hypermethylated marker has been reported. Furthermore, as the diagnosis of cardiovascular diseases is time-sensitive, it is important to develop PCR-based assays for a heart-specific marker.

Here, we applied a genomic DNA methylation sequencing-based technique, methylated CpG tandem amplification and sequencing (MCTA-seq) [5], to explore heart-specific hypermethylated markers and dynamic changes of heart-derived DNA in the blood of MI patients, and we also developed a droplet digital PCR (ddPCR) assay for detecting MI.

## Methods

### Sample collection

The study was approved by the Ethics Committee of Fuwai Hospital (Ethics No. 2018-1007). All subjects provided written informed consents for the collection of samples and subsequent analyses before inclusion in the study.

We collected tissue and plasma samples at Fuwai Hospital, Chinese Academy of Medical Science. Three pairs of left atrial and left ventricular heart tissue samples were obtained from donors who died for reasons other than cardiovascular diseases (3 males; mean age, 25.3 ± 2.1 years). Three sets of plasma samples were obtained from MI patients who were defined according to the fourth universal definition of myocardial infarction [17], with the exclusion criteria as no troponin elevation throughout the disease course, complicated with other diseases which also present with chest pain and elevated troponin such as aortic dissection or pulmonary embolism. These sets included (i) cohort 1: plasma samples obtained from patients (n = 20) after percutaneous coronary intervention (PCI), (ii) cohort 2: three series time points of plasma samples (n = 60) obtained from patients (n = 20) upon hospital admission (D0), 1 day after PCI (D1), and 2 days after PCI (D2), and (iii) cohort 3: plasma samples obtained from MI patients within 24 h of symptom onset upon hospital admission (n = 116); we also collected plasma of control individuals (n = 25), who were recruited from physical examination center of Fuwai hospital and had no history or symptoms of myocardial infarction, pulmonary embolism, aortic dissection or other significant diseases. The sample size of cohort 3 was determined using the software MedCalc (version 16.8.4). We applied MCTA-seq for cohort 1 and 2, and the *CORO6* ddPCR assay for cohort 3. All MCTA-seq results passed the quality control criterion as total molecular counts of 10,000, and all samples for the ddPCR assay were experimentally successful; thus none samples were excluded. The clinical characteristics of the patients were shown in Additional file 2: Table S1.

MCTA-seq data of nine tissues, i.e., the liver (n = 3), muscle (n = 2), lung (n = 2), stomach (n = 2), colon (n = 2), kidney (n = 2), pancreas (n = 2), skin (n = 2), and WBCs (n = 81), as well as the plasma of normal individuals (n = 202) and cancer patients (n = 229 for CRC and n = 42 for HCC), were retrieved from our previous studies [5–7].

### Blood sample processing

To obtain plasma, 4 mL peripheral blood was collected using EDTA anticoagulant tubes and the plasma samples were prepared within 6 h. The blood tube was centrifuged at 1350×*g* for 12 min at room temperature, and then the plasma was transferred to a 15-mL tube and centrifuged at 1350×*g* for 12 min, before the supernatant was transferred to a 1.5- or 2-mL tube and centrifuged at 13,500×*g* for 5 min. Finally, the plasma supernatant (approximately 2 mL) was transferred to a 1.5- or 2-mL new tube and immediately stored at − 80 °C.

### DNA extraction and library construction

Genomic DNA was extracted from WBCs and tissues using a DNeasy Blood & Tissue Kit (Qiagen, 69504) according to the manufacturer’s protocol. For MI patients and control subjects, cfDNA was extracted using a QIAamp Circulating Nucleic Acid Kit (Qiagen, 55114). For MCTA-Seq library construction, the procedures were described previously [5–7]. In brief, after bisulfite conversion (Zymo Research, D5030), cfDNA was subjected to the MCTA-Seq three-steps amplification, including (i) 1 cycle of amplification using a random primer to obtain the semi-amplicon, (ii) 1 cycle of amplification using a targeting primer characterized as having CGCGCGG at its 3′ end to obtain the full-amplicon, and (iii) 14 cycles of exponential amplification using tail primers corresponding to Illumina TrueSeq adapters (see details in Additional file 1: Methods). The final library was sequenced on an Illumina HiSeq Xten platform to generate 150-bp paired-end reads.

### Sequencing data processing

The R2 reads in FASTQ format procession were processed and filtered as previously described [5–7]. We focused on the fully methylated molecules (FMM) amplified from a CGCGCGG as the unit for calculation. The methylation value is calculated as the number of FMMs normalized by the total number of reads uniquely mapped to the whole genome, and expressed as methylated alleles per million mapped reads (MePM) for tissue samples and unique molecular identifier-adjusted MePM (uMePM) for plasma samples [5–7].

### Identification of heart-specific methylation markers

Heart-specific markers were selected by considering the MCTA-Seq methylation sequencing data of all CCGCGCGG sites within CGIs. We aimed to identify markers that give the highest signal-to-noise ratio. For a heart cfDNA methylation marker, the signal is the methylation value in the heart tissue, and the noise is the methylation level in the cfDNA. Plasma cfDNA is mainly derived from blood cells, and as we and others have previously shown, the main non-hemopoietic origin of cfDNA is the liver [7]. To this end, we focused on three parameters: the heart-to-white blood cell methylation ratio, the heart-to-plasma methylation ratio and the liver methylation value. In addition, we wanted to make sure that the signal can be released to the blood, and thus we examined whether the methylation value increase in plasma of MI patients after PCI, in which previously studies have shown that the signal from cardial cells prominently increase [14]. We consider that this increase will also indicate that the signal is derived from cardial cells but not other cell types such as fibroblast and endothelial cells in the heart tissue. The MCTA-Seq data of WBCs, normal plasma and the liver tissue were retrieved from our previous studies [5–7].

The criteria were as follows:

1. The average methylation value (MePM) in the heart tissue being 100-fold higher than that in WBCs (heart/WBC > 100);
2. The average methylation value in the heart tissue being 100 times higher than that in normal human plasma (heart/Pn > 100);
3. The average methylation value from the liver tissues being below 5 (liver < 5), as the liver has been shown to be the main nonhematopoietic source of plasma cfDNA;

The plasma from patients after PCI were used for validating that the methylation value of the loci significantly increased in comparison with the normal plasma.

### Deconvolution analysis for the heart-derived cfDNA fraction

The following equation was used to deconvolute the cfDNA tissue mapping:

$$\overline{{{\text{MP}}}} {\text{i = }}\sum\limits_{k} {\overline{MT}_{ik} *P_{k} .}$$

The deconvoluted MCTA-seq data were analyzed as previously described [7]. In this study, heart-specific markers were added to the equation. A total of 9 simultaneous equations representing 9 nonhematopoietic tissue types were generated to be solved. To further eliminate any effect from nonspecific methylation in WBCs, the average tissue fraction values in fourteen paired WBC samples (0.022%, 0, 0.28%, 0.002%, 0.019%, 0.003%, 0.2%, 0.014%, and 0.016% for the liver, lung, stomach, colon, kidney, pancreas, muscle, skin and heart, respectively) were subtracted from the measured tissue fractions. In addition, the measured tissue fractions that were lower than the average values plus three standard deviations of WBC samples (0.11%, 0, 1.62%, 0.023%, 0.0209%, 0.035%, 1.2%, 0.17%, and 0.2% for the liver, lung, stomach, colon, kidney, pancreas, muscle, skin and heart, respectively) were set to zero.

### The *CORO6* ddPCR assay

The *CORO6* ddPCR assay covered a genome region (Chr17: 27,942,532–27,942,630) located within the intragenic CGI of *CORO6*. We designed two sets of primers and probes targeting to the methylated and unmethylated alleles, respectively, which allowed simultaneously quantification the methylated and unmethylated alleles in a one tube reaction. The sequences of the two groups of primers and probes are as follows: 5′-GGGAGATTAGAATTTTTGGAGTTTAGG-3′ (forward primer), 5′-CGAAACTCGCAATCCAACCTC-3′ (reverse primer), and 5′-FAM-AGATTTACGTCGTTTTAGCG-MGB-3′ (probe), for the methylated allele; and 5′-GGGAGATTAGAATTTTTGGAGTTTAGG-3′ (forward primer), 5′-CAAATCCCAAACAAAACTCACAATCCA-3′ (reverse primer), and 5′-VIC-AGATTTATGTTGTTTTAGTGGAGGT-MGB-3′ (probe), for the unmethylated allele. For each case, cfDNA extracted from 1 to 2 mL plasma was subjected to bisulfite conversion (Zymo Research, D5030), and then the purified DNA was divided into two replicates and subjected to the ddPCR assay which were described in Additional file 1: Methods in detail.

### Bioinformatics and statistical analysis

Custom R scripts and R packages were used to construct heatmaps and to perform statistical analysis. GraphPad Prism (PRISM version 5) software was used to generate boxplots, bar plots, and AUC curves and to perform statistical analysis for the nonmultiplex tests. A P value of 0.01 or 0.05 was set as the cutoff for significance.

## Results

### Identifying heart-specific hypermethylation markers

To screen heart-specific methylation markers, we performed MCTA-seq on genomic DNA samples extracted from normal adult heart tissues (3 pairs of ventricles and atria) and cfDNA samples obtained from the plasma of MI patients after primary PCI (cohort 1, n = 20). The sequencing information are provided in Additional file 3: Table S2. We retrieved our previous MCTA-seq data of WBCs (n = 81), normal plasma (n = 202) and the liver tissue (n = 3) for searching for loci that displayed high methylation values in the heart tissue and the plasma samples after PCI, and low methylation values in normal plasma, WBCs and livers (see “Methods”) [5–7]. We also retrieved our previous MCTA-seq data of seven tissues, i.e., the muscle (n = 2), lung (n = 2), stomach (n = 2), colon (n = 2), kidney (n = 2), pancreas (n = 2) and skin (n = 2), for examining the tissue-specificity of the identified loci [7].

**Fig. 1:**
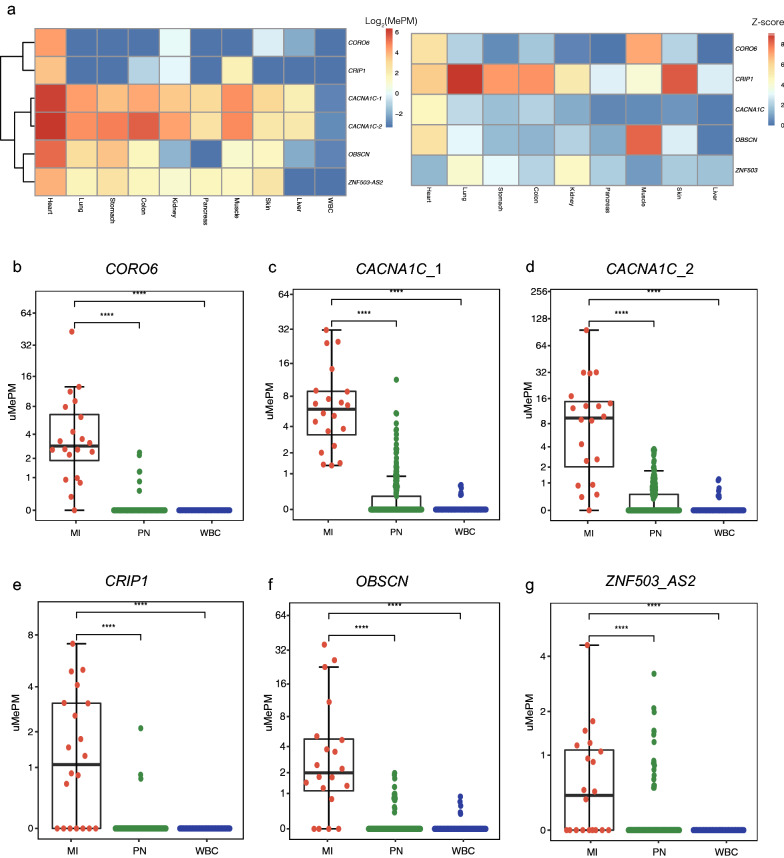
Identification of heart-specific hypermethylation markers with MCTA-seq. **a** Heatmap of 6 identified heart-specific hypermethylation marker methylation levels and the expression levels in 9 different tissues, including the heart and WBCs. Each column represents one tissue type, and each row represents a marker. The markers (n = 6) are ranked by their methylation levels in the tissue, as calculated by their MePM values of MCTA-seq. In the heatmap, blue indicates low, white and yellow indicate intermediate and red indicates high DNA methylation values, which are shown as log_2_(MePM) (left). The expression levels are shown by the log_2_(z-score) (right). See the online methods for the identification of heart-specific hypermethylation markers. **b**–**g** Comparison of the representative heart-specific marker methylation levels in the plasma of MI patients (n = 20), normal plasma (n = 202), and WBCs (n = 81). MI indicates patients with acute myocardial, PN indicates normal control individuals, and WBC indicates samples of white blood cell. ****P < 0.0001. Two-tailed MWW test

We identified six CGCGCGG loci that were located in the CpG islands (CGIs) of *CORO6*, *CACNA1C* (two loci), *OBSCN*, *CRIP1* and *ZNF503-AS2*. Among these markers, *CORO6* showed the most specific methylation pattern in the heart. Only *CORO6* showed nearly no methylation in the muscle; other loci, including another relatively specific locus, *CRIP1*, were methylated to various degrees in the muscle. The two *CACNA1C* loci had the highest methylation values in the heart, but they also showed relatively high methylation levels in other tissues, including the liver and muscle (Fig. 1a and Additional file 4: Table S3).

The methylation values of all six markers were significantly elevated in the plasma from MI patients after PCI compared with the plasma from normal individuals (P < 0.0001, two-tailed Mann–Whitney-Wilcoxon (MWW) test, Fig. 1b–g and Additional file 4: Table S3). *CORO6*, *CACNA1C-1*, *CACNA1C-2*, *OBSCN*, *CRIP1* and *ZNF503-AS2* were methylated in 95% (19/20), 100% (20/20), 95% (19/20), 80% (16/20), 65% (13/20), and 55% (11/20) of these MI patients, respectively. The two *CACNA1C* loci displayed the highest methylation values in the plasma from MI patients; however, these two markers also displayed high methylation frequencies in normal plasma (25.2%, 51 of 202 for *CACNA1C-1* and 28.7%, 58 of 202 for *CACNA1C-2*, Fig. 1c, d). *CORO6* ranked second in MI patients, and remarkably, it displayed the lowest methylation frequency in normal plasma (3.0%, 6 of 202) and WBCs (0%, 0 of 81) (Fig. 1b). *CRIP1* also displayed a low methylation frequency in normal plasma, similar to *CORO6*, but it was detected in fewer MI patients than *CORO6* (Fig. 1e). The results of the marker analysis in plasma samples were consistent with their methylation patterns in tissues.

Notably, *CACNA1C*, *CORO6* and *OBSCN* are cardiac myocyte-related genes. *CACNA1C* is a voltage-dependent calcium channel, and *OBSCN* is a component of sarcomeres [18–20]. *CORO6* is an actin-binding protein that has been shown to be highly expressed in both skeletal muscle and the heart and critical for the regulation of acetylcholine receptor clustering in skeletal muscle [21]. We confirmed the heart-enriched gene expression patterns of all three genes using the Human Protein Atlas database (Fig. 1a, right). All CGCGCGG loci were located in the intragenic region of the genes, which was consistent with our previous finding that many tissue-specific hypermethylation markers are located in the intragenic or 3′ CGIs of tissue-specific expressed genes [7].

To further evaluate the specificity of these markers, we examined the MCTA-seq data of the plasma from cancer patients retrieved from our previous studies [6, 7]. *CORO6* and *CRIP1* were barely detected in the plasma from colorectal cancer (CRC) and hepatocellular carcinoma (HCC) patients (3.9%, 9 of 229 for CRC and 9.5%, 4 of 42 for HCC), suggesting that these two markers were not hypermethylated in cancers (Additional file 1: Fig. S1 and Additional file 4: Table S3). In contrast, other markers were detected at a high frequency in cancer patients.

Together, we used MCTA-seq to identify six hypermethylation markers for detecting heart damage in the blood and *CORO6* showed the top performance.

### Dynamic changes in heart-derived DNA in MI

We next performed MCTA-seq on a second group of MI patients (cohort 2, n = 20), from whom serial plasma samples were collected at three time points: at hospital admission before PCI (D0), 1 day after PCI (D1), and 2 days after PCI (D2). Sequencing information of theses samples are provided in Additional file 3: Table S2.

**Fig. 2:**
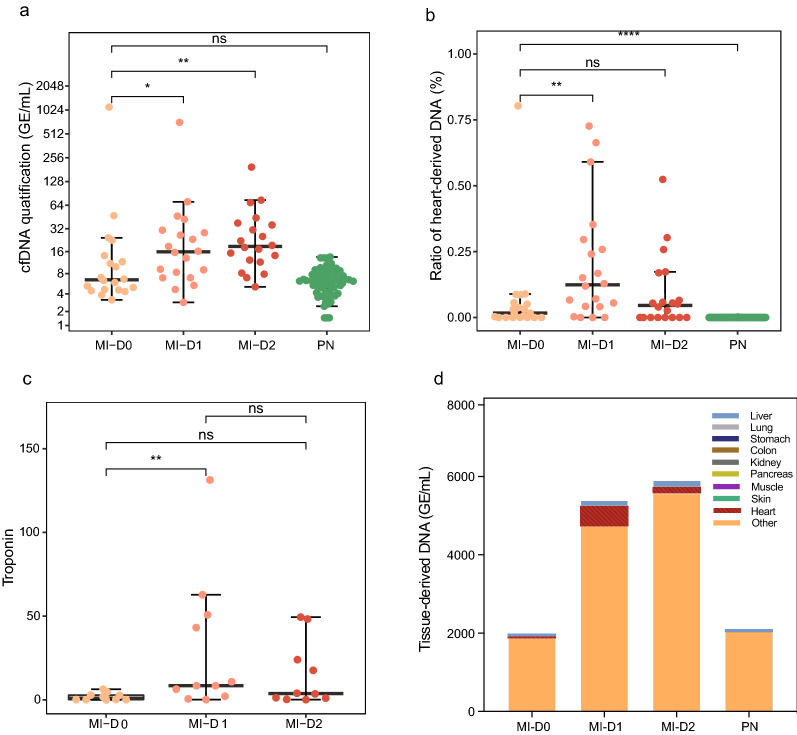
Comparisons of cfDNA and Tn levels in MI patients and normal individuals. **a** Comparison of cfDNA concentration (ng/mL plasma) in MI patients and normal individuals. **b** Ratio of heart-derived DNA in MI patients and normal individuals. **c** Tn levels in MI patients at different times. **d** Contribution of different tissues to the plasma of MI patients and normal individuals. D0, D1, D2 indicate MI upon hospital admission before PCI, 1 day after PCI, and 2 days after PCI, respectively. n = 20 for D0/D1/D2 samples and 67 for control individuals. *P < 0.05, **P < 0.01, ****P < 0.0001; ns, no significant difference. Two-tailed MWW test. The statistical values are the median

The concentration of cfDNA was similar in MI patients at admission and normal individuals (paired two-tailed MWW test, P = 0.21, median 6.5 ng/mL for D0 MI patients and 6.33 ng/mL for the normal individuals, Fig. 2a). Notably, the concentration significantly increased at 1 or 2 days after PCI compared with at admission (median 15.9 ng/mL and 18.8 ng/mL for D1 and D2 cases, respectively, paired two-tailed MWW test, P = 0.02395 for D1 vs. D0 and P = 0.03623 for D2 vs. D0); no significant difference was found between D1 and D2 (paired two-tailed MWW test, P = 0.67) (Fig. 2a and Additional file 5: Table S4). These results were consistent with the previous study showing that the concentration of cfDNA peaks after PCI [16].

We investigated the tissue of origin of the increased cfDNA after PCI. We extended our previously reported deconvolution approach to infer the tissue fractions of the heart and eight other nonhematopoietic tissues (see “Methods”). Notably, the results showed that heart-derived DNA was significantly elevated in the plasma from MI patients at admission compared with the controls (median 1.6% for MI versus 0% for control, P = 1.0168E−11, Fig. 2b). The fraction of heart-derived DNA was clearly elevated on the first day after PCI, while it significantly decreased on the second day after PCI (median 12% and 0.4% for D1 and D2, respectively, Fig. 2b). The level of high-sensitivity troponin (hs-cTn) showed a similar dynamic pattern (median 1.05 for D0 versus 8.46 for D1, P = 0.003652); 3.77 for D2 versus 8.46 for D1, P = 0.3144, Fig. 2c and Additional file 5: Table S4). These dynamic changes were consistent with Zemmour et al.’s study and indicated that MCTA-seq detected true signals of heart injury [14]. Examination of the relationship between the fraction of heart-derived DNA and high-sensitivity troponin showed a correlation coefficient of 0.48 (Additional file 1: Fig. S2).

**Fig. 3:**
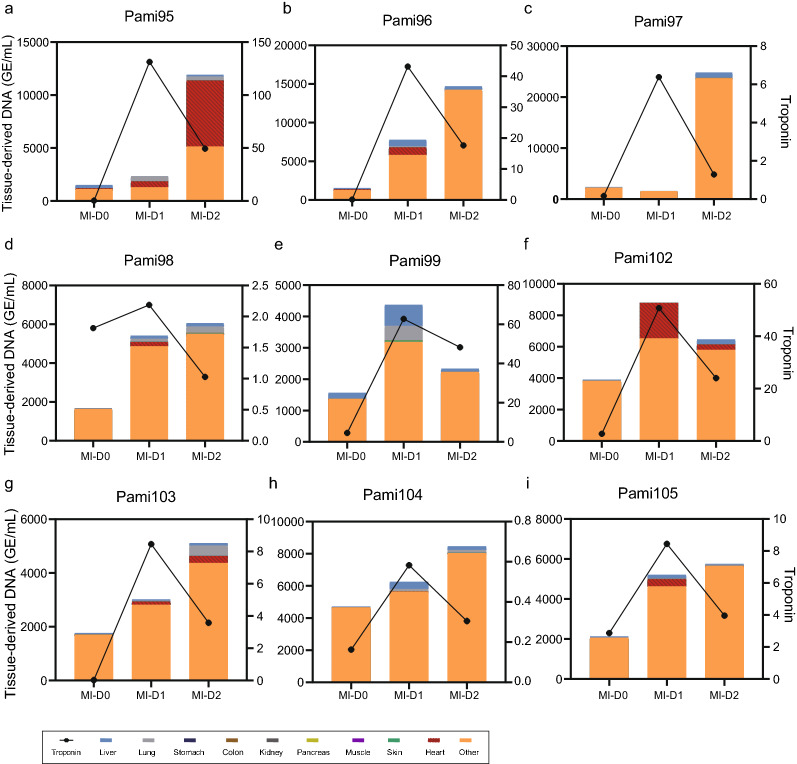
Dynamic changes in different tissue-derived DNA and Tn levels during MI and after PCI in representative individual patients. Contribution of different tissues to plasma cfDNA and Tn levels in MI patients based on MCTA-seq deconvolution analysis. D0, D1, D2 indicate MI upon hospital admission before PCI, 1 day after PCI, and 2 days after PCI, respectively. n = 20 for D0/D1/D2 samples. The statistical values are the median. Different colors in the bar graph indicate the contribution of different tissues, and the zig–zag line indicates the change in troponin

The data revealed a discordance between the cfDNA concentration and the heart fraction on the second day after PCI: the total cfDNA concentration remained high while the heart fraction decreased (Fig. 2d). Deconvolution analysis showed that the increased cfDNA at D2 was mainly derived from blood cells (Fig. 2d). Also, among the 3130 increased cfDNA counts from D0 to D1 (median values: 2170 and 5300 GE/mL for D0 and D1, respectively), only approximately 20% (median 512 GE/mL) were derived from the heart. The heart-derived DNA amount clearly decreased to a median of 159 GE/mL at D2 (Fig. 2d and Additional file 5: Table S4). The pattern of dynamic changes was confirmed in individual patients (Fig. 3a–i and Additional file 1: Fig. S3). However, there were also exceptions. For example, both the total and heart-derived cfDNA amounts clearly increased in the D2 plasma of patient Pami95, although the hs-cTn level decreased (Fig. 3a); the peak hs-cTn level of that patient was extraordinarily high, suggesting severe heart damage.

Together, these results showed that heart-derived DNA increased in the plasma of MI patients both before and after PCI, while the surge in total cfDNA concentration after PCI was mainly derived from blood cells.

### A ddPCR assay for detecting MI

**Fig. 4:**
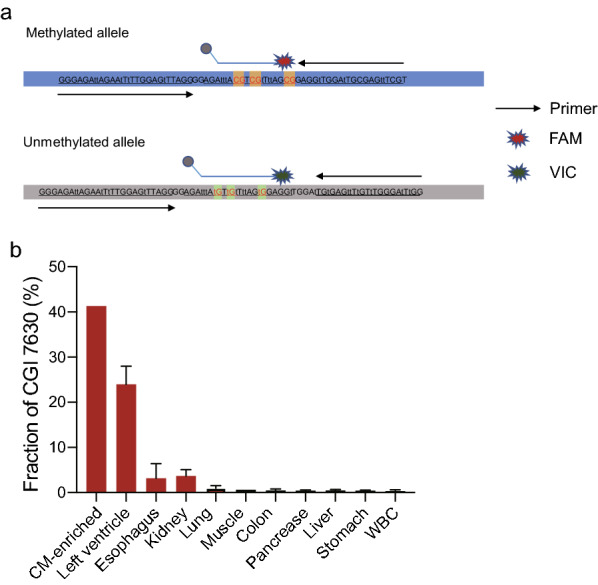
Detection of MI using ddPCR. **a** Schematic of the approach for the ddPCR-based detection of the methylation status of three CpG sites in the reverse strand of *CORO6*. **b** The methylation of the *CORO6* locus in different tissues, enriched cardiomyocytes and WBCs. CM indicates cardiomyocytes, WBC indicates white blood cell

Among the six identified heart methylation markers, the *CORO6* locus showed the best heart specificity and lowest frequency in normal plasma. We therefore explored the development of a ddPCR assay for this locus. Two pairs of primers were designed to amplify the methylated and unmethylated states of a 71-bp region (Fig. 4a). Two TaqMan probes were designed to detect three common CpG sites within the amplicon, with a FAM probe for the methylated amplicon and a VIC probe for the unmethylated amplicon (Fig. 4a). A single-tube reaction distinguished the signals of the methylated and unmethylated amplicons.

We first used the assay to examine tissue samples, including the heart, esophagus, kidney, lung, muscle, colon, pancreas, liver, stomach and WBCs. For the heart, methylated molecules accounted for 23% of all amplicons. In contrast, the ratios were 0.79%, 0.39% and 0.015% for the muscle, liver, and WBCs, respectively; slight ratios of 3.61% and 3.14% were detected in kidney and esophagus, respectively (Fig. 4b). To investigate whether the signal of *CORO6* was from cardiomyocytes, we enriched cardiomyocytes from a heart tissue sample obtained from human myocardial hypertrophy (HCM) surgery. The *CORO6* signal increased to 40% in the cardiomyocyte-enriched portion and remained at 24% in the unenriched portion, suggesting that hypermethylation of *CORO6* was cardiomyocyte-specific (Fig. 4b). It was notable that *CORO6* gave high heart:WBC and heart:liver ratios, which are two of main sources of cfDNA [7]. The heart:muscle signal ratio was also high, which should be useful for distinguishing between heart and muscle diseases.

**Fig. 5:**
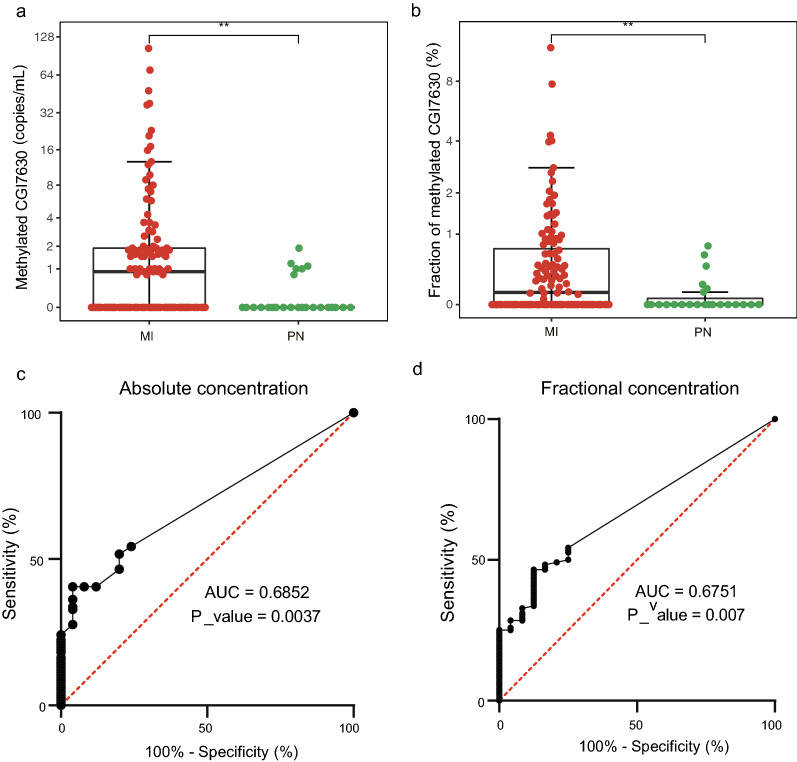
The results of the ddPCR assay for detecting MI. **a** Measurement of the absolute concentration of methylated molecules of the *CORO6* locus in the plasma of MI patients and control individuals. **b** Comparison of the fractional concentration between MI patients and control individuals. **c** Receiver operating characteristic (ROC) curve for the diagnosis of MI by the absolute concentration in plasma of MI patients and control individuals. **d** Receiver operating characteristic (ROC) curve for the diagnosis of MI based on the fractional concentration in the plasma of MI patients (n = 116) and control individuals (n = 25). **P < 0.01. Two-tailed MWW test

Then, we applied the assay to plasma samples from 116 MI patients and 25 control individuals. All plasma samples from MI patients were collected before PCI and within 24 h of the onset of chest pain upon hospital admission. The results showed that the *CORO6* methylation signal was significantly higher in MI patients than in controls (median 0.99 [interquartile range (IQR) 0.77–1.98] vs. 0 [IQR: 0–0.91] copies/mL; P = 0.001861) (Fig. 5a and Additional file 6: Table S5). The methylation signal was detected in 54 of 116 MI patients, ranging from 1 to 104 copies/mL, while in contrast, it was detected in 20% (5 of 25) of controls at 1 or 2 copies/mL. The fractional concentration in MI patients was also significantly higher than that in controls (P = 0.005703, Fig. 5b and Additional file 6: Table S5). The area under the curve (AUC) values were 0.6852 (95% confidence interval (CI) 0.59–0.78, P = 0.0037) and 0.6751 (95% CI 0.57–0.78, P = 0.007) for the absolute concentration and for the fractional concentration, respectively (Fig. 5c, d). When one copy of cardiac-specific cfDNA/mL was defined as the cutoff for a positive signal, the diagnostic sensitivity was 46%, and the specificity was 80%. When 0.2% cardiac-specific cfDNA/mL was defined as the cutoff for a positive signal, the diagnostic sensitivity was 47%, and the specificity was 84%.

In summary, we established a methylated *CORO6* ddPCR assay for the detection of heart-derived DNA in the blood.

## Discussion

In this study, we conducted MCTA-Seq to identify heart-specific methylated markers and investigated the origin and dynamics of the increased cfDNA in MI patients. Among the identified markers, *CORO6* shows the top performance. We developed a *CORO6* ddPCR assay for detecting heart damage in blood.

MCTA-seq is suitable for screening cfDNA methylation markers since it detects thousands of hypermethylated CGIs in cfDNA in a semi-targeted manner. Among the detected CGIs, the *CORO6* locus emerged as the best heart-specific hypermethylation marker. The *CORO6* ddPCR assay detected approximate 20% methylation level in the heart and 0.015% in WBCs. As the heart tissue is composed of approximately 30% cardiomyocytes [22], the ratio is estimated to be approximate 60% in cardiomyocytes. Zemmour et al. [14] have previously described unmethylated FAM101A as the first heart-specific marker. Methylated *CORO6* was detected in a similar percentage of control individuals compared with unmethylated FAM101A (29% for the FAM101A sequencing-based assay and 20% for the *CORO6* ddPCR assay), indicating that the two loci have similar background levels in the blood. The signal of FAM101A is higher than that of *CORO6* in the cardiomyocytes (89% for FAM101A and ~ 60% for *CORO6*). However, the amplicon length of the *CORO6* ddPCR assay (71 bp) was shorter than that of the FAM101A sequencing-based assay (90 to 100 bp). Since cfDNA is highly fragmented and bisulfite treatment further reduces the length, a short amplicon should give a higher signal than a long amplicon, particularly for cfDNA detection. FAM101A sequencing-based assay has shown an AUC value of 0.76 for detecting plasma before PCI, and the *CORO6* dd*PCR* assay showed an AUC value of 0.68 (95% CI 0.59–0.78). We consider that the performance of the *CORO6* assay is comparable to that of the FAM101A sequencing-based assay for detecting heart-derived DNA.

An advantage of the *CORO6* ddPCR assay is that it is more rapid and convenient than the FMA101A sequencing-based assay. Zemmour et al. also developed a ddPCR assay for FAM101A. However, since the marker requires the simultaneous interrogation of six CpG sites crossing a relatively long distance, it is not possible to perform a standard ddPCR assay. Though the authors cleverly used two fluorescent probes to cover five CpG sites, the technical specificity of the ddPCR assay is still approximately 50-fold worse to the sequencing-based assay; thus, the performance of the FAM101A ddPCR assay is not satisfactory. In contrast, the *CORO6* assay showed high specificity comparable to the FAM101A sequencing-based assay, with a typical ddPCR design that interrogates 3 CpG sites using one 20–25 bp TaqMan probe. In normal plasma, the FAM101A ddPCR assay has been reported to show a specificity of 53%, while the *CORO6* ddPCR assay showed a specificity of 80% [23]. In addition, comparing with a hypomethylation marker, a hypermethylation marker provides a technical advantage as relatively resisting to contamination from the unmethylated amplified PCR products, which are converted into unamplifiable products by the bisulfite treatment. The performance of the *CORO6* ddPCR assay may be further increased by optimizing the primer and probe, and by improvement of DNA methylation detection technique.

The *CORO6* ddPCR assay provides a simple method for investigating clinical situation with heart injure. Our study provides the first independent experimental validation of Zemmour et al.’s study showing the release of cardiac-derived cfDNA during MI [14]. A recent report has shown elevation of cardiomyocyte-specific cfDNA in heart failure patients using the FMA101A ddPCR assay. The elevation of the total cfDNA level has been shown in uncontrolled hypertension; yet the source remains to be determined [23]. Future investigation is needed for the usage of the heart-specific methylation marker in MI, heart failure and hypertension. In addition, the heart-specific methylation marker including the *CORO6* ddPCR assay may complement the genetic and sequencing-based cfDNA method for monitoring heart transplantation, which is quicker and able to distinguish between cardiac and coronary released donor cfDNA [4]. It is also possible to further increase the specificity and sensitivity of the *CORO6* assay by testing regions adjacent to the one covered by our present ddPCR assay, by adding the antisense information, or by combining FAM101A or other types of molecules such as miR-208 and miR-499 [24–27].

We showed that only a small portion of the increased cfDNA was derived from the heart in MI patients who underwent PCI. We made a similar finding in acute pancreatitis patients in whom the increased cfDNA was also mainly not derived from the pancreas [7]. Recently, Moss et al. more precisely showed that the increased cfDNA in sepsis patients is mainly derived from granulocytes [13]. Thus, it appears that elevation of cfDNA from WBCs is common in acute clinical situation and reflects an immune response. It is interesting that the total WBC count has been associated with the risk of coronary heart disease [28], and an increase in the WBC count after an MI episode has been shown to be a predictor of worse patient prognosis [29]. Increased cfDNA levels have also been shown to be a prognostic marker in a small cohort of MI patients [30]. Distinguishing tissue of origin of the elevated cfDNA may provide more information for prognosis prediction.

## Conclusions

Our comprehensive cfDNA methylation analysis not only provides insights into the source of the increased cfDNA relating to cardiac pathologies of MI, but also identified heart-specific methylation markers. The *CORO6* ddPCR assay may be useful for investigation of myocardial damage in clinical applications.

## Supplementary Information

**Additional file 1.** Methods. **Figure S1.** Comparison of heart-specific marker methylation levels in the plasma of CRC and HCC patients and WBCs. n = 229, 42, 81for CRC and HCC patients and WBCs. *P < 0.05, **P < 0.01, ***P < 0.001, ****P < 0.00001; ns, no significant difference. Two-tailed MWW test. **Figure S2.** Correlation between ratio of heart-specific cfDNA and Tn levels in patients with MI. **Figure S3.** Dynamic changes in different tissue-derived DNA and Tn levels during MI and after PCI.

**Additional file 2: Table S1.** Baseline characteristics of study cohort.

**Additional file 3: Table S2.** Sequencing information.

**Additional file 4: Table S3.** Methylation values (MePM) of 6 heart specific markers in tissue and plasma samples.

**Additional file 5: Table S4.** Total cfDNA concentrations, contribution from different tissues and levels of Tn in cohort2.

**Additional file 6: Table S5.** Results of ddPCR assay.

## Data Availability

The raw sequencing data were deposited in The Genome Sequence Archive for Human (GSA-Human) with the accession number HRA000358.

## Abbreviations

MI: Myocardial infarction
PN: Plasma of normal control individuals
cfDNA: Circulating cell-free DNA
MCTA-seq: Methylated CpG tandem amplification and sequencing
ddPCR: Digital droplet PCR
PCI: Percutaneous coronary intervention
HCC: Hepatocellular carcinoma
CRC: Colorectal cancer
WBC: White blood cell
