# Supplementary material for: Heart-specific DNA methylation analysis in plasma for the investigation of myocardial damage
Source: J Transl Med. 2022 Jan 21;20:36. doi: 10.1186/s12967-022-03234-9 (PMC8780310; doi:10.1186/s12967-022-03234-9)
Supplement: Supplementary file 1 — Additional file 1. Methods. Figure S1. Comparison of heart-specific marker methylation levels in the plasma of CRC and HCC patients and WBCs. n = 229, 42, 81for CRC and HCC patients and WBCs. *P < 0.05, **P < 0.01, ***P < 0.001, ****P < 0.00001; ns, no significant difference. Two-tailed MWW test. Figure S2. Correlation between ratio of heart-specific cfDNA and Tn levels in patients with MI. Figure S3. Dynamic changes in different tissue-derived DNA and Tn levels during MI and after PCI. [file 12967_2022_3234_MOESM1_ESM.docx]

**Methods**

**MCTA-seq library construction**

Briefly, cfDNA from 2 mL plasma was bisulfite treated with EZ-96 DNA Methylation-Direct™ MagPrep (Zymo Research, D5030). All bisulfite-converted cfDNA was subjected to MCTA-seq library preparation. First, all bisulfite-converted DNA was linearly amplified in a 15-µL reaction containing 1*NEB buffer 2 (NEB, B7002S), 250 µM of each dNTP, 0.33 µM MCTA-seq primer A (a mixture of four primers: (i) 5′-TTTCCCTACACGACGCTCTTCCGATCTHHHHHHHHCGCH-3′, (ii) 5′-TTTCCCTACACGACGCTCTTCCGATCTHHHHHHHCGHCH-3′, (iii) 5′-TTTCCCTACACGACGCTCTTCCGATCTHHHHHHCGHHCH-3′, and (iv) 5′-TTTCCCTACACGACGCTCTTCCGATCTHHHHHCGHHHCH-3′; the underlined portions correspond to the UMI sequences (H = A/T/C)). and 2.5 units Klenow fragment (NEB, M0212) with no 3’ to 5’ exonuclease activity to obtain the semi amplicon. The reaction was assembled except for the Klenow fragment and incubated at 95 °C for 2 min before holding at 4 °C. The Klenow fragment was then added. Then, the reaction mixture was subjected to the following conditions: 4 °C for 50 s, 10 °C for 1 min, 20 °C for 4 min, 30 °C for 4 min, 37 °C for 4 min and 75 °C for 20 min (to inactivate the Klenow fragment). In the second step, by adding a 5-µL mixture containing 1*E* Taq buffer, 1.5 units Hot Start E*Taq (Takara, RR006B), and 1 µM MCTA-seq primer B (5'-GTGACTGGAGTTCAGACGTGTGCTCTTCCGATCTDDDDCGCGCGG-3', D = A/T/G), enriched CpG regions were selected and amplified in a 20-µL reaction. The reaction mixture was subjected to the following conditions: 95 °C for 3 min, followed by 50 °C for 2 min and 72 °C for 1 min. Then, the full amplicon was amplified using index primers C and D in a total reaction volume of 50 µL by adding 30 μL of solution containing 1× Ex Taq Buffer, 250 μM of each dNTP, 2 μM primer C (5'-AATGATACGGCGACCACCGAGATCTACACTCTTTCCCTACACGACGCTCTTCCGATCT-3') and 2 μM primer D (5'-CAAGCAGAAGACGGCATACGAGATCTGATCGTGACTGGAGTTCAGACGTGTGCT-3'. The underlined portion in primer D corresponds to the Illumina index sequence (the sequence corresponding to index 9 is shown here), and the reaction mixture was subjected to 14 cycles of 95 °C for 30 s, 65 °C for 30 s, 72 °C for 1 min and a final cycle of 72 °C for 5 min. After the third amplification step, we pooled six samples with different Illumina index sequences by taking 30-μL reactions for each sample, and then purified the samples to concentrate their volume using a DNA Clean & Concentrator™-5 Kit (Zymo research, D4033). The resulting product was resolved on a 3% agarose gel (Takara, Agarose LM SIEVE, D614), and the 180-250 bp fraction was excised and then purified. Plasma samples usually needed to be subjected to two additional rounds of amplification (using primers QP1 (5'-AATGATACGGCGACCACCGA-3') and QP2 (5'-CAAGCAGAAGACGGCATACGA-3')) and gel purification to remove primer dimers and acquire enough material for sequencing.

**The *CORO6* ddPCR assay**

First, twenty microliters of each reaction mix were prepared, which consisted of 10 µL ddPCR Supermix for Probes (no dUTPs) (Bio-Rad), 9.6 µL of bisulfite-converted DNA, a final concentration of 450 nM of each forward primer and reverse primer, and 250 nM of non-methylation-specific and methylation-specific probe. Then, 20 µL of reaction mixture and 70 µL of droplet generation oil (Bio-Rad) were loaded on a QX200 ddPCR droplet generator (Bio-Rad). Next, a droplet containing one or no template was carefully transferred into a 96-well PCR plate and sealed immediately. For each run, fully methylated human genomic DNA (FMG, EMD Millipore) was used as a positive control, and no template was used as a negative control.

PCR was performed on a thermal cycler, and the program consisted of 95 °C for 10 min followed by 40 cycles of 94 °C for 15 s and 60 °C for 1 min and a final inactivation incubation at 98 °C for 10 min. After PCR, the plate was transferred to a QX200 droplet reader (Bio-Rad), and droplets from each sample were analyzed using QuantaSoft (version 1.7) software (Bio-Rad). The cutoff values for positive fluorescence signals in the FAM and VIC channels were determined according to the positive controls and the WBC control. After analysis, we obtained the copies of the methylated and unmethylated molecules in each sample.

**Fig. S1: Comparison of heart-specific marker methylation levels in the plasma of CRC and HCC patients and WBCs.** n = 229, 42, 81 for CRC and HCC patients and WBCs, respectively. *P < 0.05, **P < 0.01, ***P < 0.001, ****P < 0.00001; ns, no significant difference. Two-tailed MWW test.

**Fig. S2: Correlation between ratio of heart-specific cfDNA and Tn levels in patients with MI.**

**Fig. S3: Dynamic changes in different tissue-derived DNA and Tn levels during MI and after PCI.**Contribution of different tissues to plasma cfDNA and Tn levels in the remaining individual MI patients based on MCTA-seq deconvolution analysis. D0, D1, D2 indicate MI upon hospital admission before PCI, one day after PCI, and two days after PCI, respectively. n = 20 for D0/D1/D2 samples. The statistical values are the median. Different colors in the bar graph indicate the contribution of different tissues, and the folded line indicates the change in troponin.
